# Tuberculosis

**Published:** 1904-09-10

**Authors:** 


(*To be continued.*)

---

### TUBERCULOSIS.

**Early Diagnosis.**—The importance of the history of the patient is shown by a series of cases described by Batty Shaw[1] of apical bronchiectasis. In these the condition had lasted for years without loss of weight, and all examinations of the sputum had proved negative. Owing, perhaps, to better drainage of apical bronchiectatic cavities, the typical offensive expectoration was absent. Shaw cautions practitioners, however, against diagnosing apical bronchiectasis till after a thorough examination of the sputum and the application of the tuberculin test. A history of pleurisy is considered as very suggestive of tubercle by Cattle.[2] Baumler[3] insists on the importance of the general symptoms, continued fever, lassitude, pallor, slight shiverings, night sweats, loss of appetite and weight without physical signs and even without cough. In children, where the spleen may be slightly enlarged, these cases must be distinguished from mild typhoid by a bacteriological examination of the blood, Widal's test, and the leucocyte count. The diazo-reaction in the urine may occur with either condition. Hæmoptysis, due to tuberculosis, must be distinguished from that seen in mitral stenosis (often unaccompanied by a *bruit*), especially as fine crepitations along the border of the left lung near the heart are sometimes heard in the latter disease. Repeated careful examination of the heart in the recumbent position should clear up the diagnosis, and the absence of fever is of assistance. Cattle observes that the majority of cases of slight hæmoptysis, even without physical signs, are tuberculous, but that bleeding from the upper respiratory passages, stomach, and pharynx must of course be carefully excluded. Baumler notes hypertrophy of the tonsils and slight pharyngeal catarrh, enlargement of the thyroid causing local congestion by pressure on the trachea, and aneurysm of the aorta pressing on the trachea or a bronchus as liable to cause confusion, especially as the last two conditions may cause physical signs in the lungs. Owen[4] states that hæmoptysis may be the first symptom to which the patient's attention is called, even though there are frequently physical signs in the chest by that time. A history of recurrent hæmoptysis is especially valuable, owing to the intermittent advances of the disease in its earlier phases. The examination of the sputum, if positive, is a valuable aid, but bacilli are generally absent in early cases, and repeated examinations are necessary. Cattle quotes a case of Latham's in which the ninth bacteriological examination was the first which showed bacilli, six slides having been prepared on each occasion. Owen and Cattle insist that valuable time should not be lost in waiting for the appearance of bacilli, but the former states that they may sometimes be found in the fauces when there is no expectoration. Batty Shaw would only exclude tuberculosis when the bacilli were not demonstrated after examinations frequently repeated during some years. As regards physical signs, Owen and Baumler remark that the auscultatory sounds are often more characteristic than dulness or resonance; and the former advises that, after a cursory inspection of the chest, auscultation should at once be proceeded with and percussion left till later. Owen, Baumler, and Cattle note the error which may arise if the fact is forgotten that the normal right apex often shows impaired resonance with louder breath and voice sounds, and more marked tactile vocal fremitus. Baumler also points out that lateral curvature or kyphosis may considerably modify the percussion note at the apex, and displace the heart where no disease of the lungs is present. Owen states that superficial consolidation must extend about half an inch into the lung to give conduction to tubular breath sounds. Deeper consolidation not reaching the surface may give "distant" bronchial breathing, and a chain of consolidated points will give "harsh" breathing. Mere prolongation of the expiratory sound may be produced by slight catarrhal changes in the tubes or emphysema, and is normal in children. The pitch of tubular breathing in phthisis is much lower than in pneumonia, and in this connection Batty Shaw quotes two cases of subacute apical pneumonia, in which the diagnosis rested on the rapid and complete recovery of the patient and the number of pneumococci found in the sputum. "Cogwheel," or "wavy," breathing is attributed by Owen to rather extensive deep consolidation not reaching to the surface, and Baumler says it may be heard round the edges of a consolidated area, and is probably due to narrowing of the tubes. Owen states that bronchophony *plus* tubular breathing indicates extensive consolidation; apart from tubular breathing it merely shows that the lesion is deep-seated; tubular breathing without dulness is caused by compensatory emphysema due to a fibrous scar, a condition which may give rise to physical signs which are very puzzling on account of their rapid variations. Dulness to light percussion without tubular breathing is probably due to a thickened pleura; if, however, the dulness is only obtained on "deep" percussion, it may be caused by a deeply seated patch of consolidation; when scattered tubercles are present the dulness will appear with either "deep" or "light" percussion. The important adventitious sounds, according to Owen, are fine inspiratory crepitations, due to œdematous infiltration of the alveoli, and the sharp sticky click at the end of inspiration due to inflammatory changes. The latter heard at the *edges* of a patch show advancing lesions, *over* a patch they probably denote disintegration. Both these sounds are high pitched and localised to the apices, thus differing from ordinary bronchitic râles. Dry wheezing rhonchi limited to the apex and constantly heard are also characteristic, and are heard often in the absence of râles owing to their higher conductivity. In the absence of other physical signs limited expansion and flattening of the apex with decreased girth may show an old fibrous lesion or broncho pneumonic active disease. The diagnosis must be made by the general symptoms, but the patient must be observed for at least a month.

Baumler notes that often the râles can only be heard over the upper part of the interscapular space, more often on the right side, and says that in broncho-pneumonic cases secondary foci should be sought in the second and third intercostal spaces towards the axilla. In older persons subject to bronchitis, Owen attaches importance to a change in the character of the rhonchi as indicating the onset of tuberculosis. In these cases the rhonchus becomes "broken," the latter part sounding high pitched. The diagnosis of cavitation can only be made when the cavity is covered by solid and not by emphysematous tissue. In these cases small cavities may be detected by the expiratory sound being higher pitched than the inspiratory; pectoriloquy and the post-vocal "whiff" are of little value in the diagnosis of small cavities. Baumler notes that the tympanitic resonance heard over a cavity may also be heard over completely consolidated lung, as also may the "cracked-pot sound" and Wintrich's sign (change of pitch occurring when the mouth is shut or opened). But the auscultatory sounds over solid and excavated lung differ. The tuberculin test is strongly advocated by Baumler and Freudenthal,[5] and is also considered useful by Cattle and Owen; cautiously used, these writers do not consider it dangerous. Owen and Cattle regard skiagrams as useful merely to confirm the diagnosis as made by physical examination. Special diagnostic signs of interest are the rapid pulse (Cattle), and the pallor of the hard and soft palate on either side of the uvula and the epiglottis as contrasted with the inflamed condition of adjacent parts (Freudenthal). The latter also calls attention to the frequency with which phthisis is preceded by atrophic rhinitis, and states that Alexander also found ozæna frequently accompanied by latent lung disease. Cattle notes that the lesions in phthisis may begin at the apices of the lower lobes and extend down the septum, *i.e.*, from a point level with the third dorsal spine and running down the inner border of the scapula when the elbow is raised and the hand placed over the opposite shoulder. Baumler, holding that the disease often begins as a bronchitis of the larger tubes in the upper lobe, states that it may spread and become apparent either by direct extension or by conduction to the areas supplied by the tubes. All writers condemn the use of the term "stages of consumption." These do not exist clinically, as, though pathologically the three stages of consolidation, softening, and cavitation may be distinguished, they are all generally present at the same time in different parts of the lung.

[1] Clin. Jour., March 30, 1904, p. 377. [2] Pract., February 1904, p. 218. [3] Brit. Med. Jour., April 2, 1904, p. 769. [4] Ibid., April 2, 1904, p. 765. [5] Med. Rec., March 12, 1904, p. 409.

(*To be continued.*)

